# Nutritional value of *Sesamum indicum* L. was improved by *Azospirillum* and *Azotobacter* under low input of NP fertilizers

**DOI:** 10.1186/s12870-019-2077-3

**Published:** 2019-11-04

**Authors:** Asia Nosheen, Asghari Bano, Rabia Naz, Humaira Yasmin, Ishtiaq Hussain, Faizan Ullah, Rumana Keyani, Muhammad Nadeem Hassan, Ayesha T. Tahir

**Affiliations:** 1 Department of Biosciences, COMSATS University, Park Road, Chak Shahzad, Islamabad, 44000 Pakistan; 2 Department of Biosciences, University of Wah, Wah Cantt, Pakistan; 3 Department of Agriculture Research, Biotechnological Research and Development Section, Tissue Culture Lab, Gilgit-Baltistan, Pakistan; 4 Department of Botany, University of Science and Technology, Bannu, KPK Pakistan

**Keywords:** Sesame, *Azospirillum*, *Azotobacter*, Fatty acid, Oil quality, Nutritional value, Chromatography, Oleic acid

## Abstract

**Background:**

Sesame (*Sesame indicum* L.) is well-known as a versatile industrial crop having various usages and contains 50–55% oil, 20% protein, 14–20% carbohydrate and 2–3% fiber. Several environmental factors are known to adversely affect yield and productivity of sesame. Our overall aim was to improve the growth, yield and quality of sesame cv. TS-3 using plant growth promoting rhizobacteria (PGPR) and saving the nitrogen and phosphate fertilizers (NP) by 50%. Field experiment (randomized complete block design) was conducted during the months of July to October of two consecutive years 2012–2013. *Azospirillum* (AL) and *Azotobacter* (AV) were applied as seed inoculation alone as well as along with half of the recommended dose of nitrogen (N) and phosphate (P) fertilizers (urea and diammonium phosphate) at the rate of 25 kg/ha and 30 kg/ha respectively.

**Results:**

Here we report that *A. lipoferum* along with half dose of NP fertilizers (ALCF) were highly effective in increasing the agronomic and yield traits of sesame as compared to the control. *A. vinelandii* plus NP fertilizers (AVCF) exhibited higher seed oil content. Minimum acid value, optimum specific gravity and modified fatty acid composition were observed in ALCF treatment. Increase in oleic acid by ALCF is directly linked with improved oil quality for health benefits as oleic acid is the fatty acid which creates a balance between saturation and unsaturation of oil and for the hypotensive (blood pressure reducing) effects.

**Conclusion:**

It is inferred that ALCF treatment improved plant growth, seed yield and oil quality of sesame pertaining to good quality edible oil production.

## Background

Vegetable oil has an important role in our food and is the essential component of human health. Global demand for vegetable oils is growing and estimated to reach 240 million tons by 2050 [1]. In Asian countries, sesame seeds as a source of health food have been used for disease prevention for several thousand years. It was reported that sesame contains such compounds that benefit to human health, which include antioxidant, anticancer, antiaging, cholesterol lowering, antihypertensive and antimutagenic properties [2]. Sesame oil contains the most abundant fatty acids i.e. linoleic, palmitic, and stearic acids, which together comprised about 96% of the total fatty acids [3]. The demand for edible oil is increasing gradually with the increase of world population, therefore, cultivation of oil seed crops is expending all over the world [4]. However, efforts are needed to increase the production of vegetable oils by environmental friendly and cost-effective measures. Sustainable measures are being explored to improve the quality and quantity of edible oil for human consumption because excessive utilization of chemical fertilizers has adverse effects on environment and is one of the major causes of soil and water pollution. Biofertilizers supplied in combination with chemical fertilizers could minimize the aforementioned problems [5, 6]. Biofertilizers mainly include those microorganisms which have capability to fix atmospheric nitrogen, release plant growth promoting substances and solubilize rock phosphates etc. These microorganisms include fungi such as Arbuscular Mychorrhizal (AM) and bacteria like plant growth-promoting rhizobacteria (PGPR). These microorganisms promote plant growth and yield independently as well as synergizing the effect of each other.

The utilization of PGPR as biofertilizers is increasingly being reported as a way of complementing conventional inputs in agricultural systems [7].

The PGPR are present naturally in the soil and improve plant growth, immunity and productivity [8]. Shakeri et al. [9] and El-Habbasha et al. [10] reported that application of PGPR in combination with half and quarter dose of chemical fertilizers resulted in improved agronomic attributes, yield and yield components as well as seed and protein contents of sesame over the other treatments and uninoculated plants [11]. They further reported that PGPR treatment increased oleic acid contents by reducing the palmitic and stearic acid contents. Similarly, previous studies reported that application of PGPR improved leaf area index, dry matter, harvest index, yield and yield components, seed oil contents and protein contents in canola and sesame [12, 13]. Thus, PGPR are ideal bioinoculants for increasing the yield as well as quality of edible crops. The diversity in rhizobacteria due to genetic drift mediated by continuous changing climate has necessitated the evaluation of new strains on different cultivars and environmental conditions.

Sesame is one of the important industrial crops and its oil is rich in nutrition. The present investigation was aimed to augment the growth, yield and oil quality of sesame (*Sesamum indicum* L.) with the perspective to edible oil production by the application of *Azospirillum* and *Azotobacter* to minimize the use of nitrogen and phosphate fertilizers for green and sustainable agriculture.

## Results

The soil analysis of the field was also carried out and shown in the Table 1.

**Table 1:** Soil analysis

Physicochemical properties	2012	2013
Soil Texture	Sandy clay loam	Sandy clay loam
E.C (dSm^−1^)	0.33	0.36
pH	7.42	7.31
Total nitrogen (%)	0.043	0.047
Soil Organic matter (%)	0.69	0.71
Available phosphorous	3.46	3.40
Ca^2+^	10.31	11.17
K^+^	17.15	20.30
Mg^2+^	2.49	2.90
Na^+^	7.71	7.80
Fe^2+^	3.25	3.57
Cu^2+^	0. 50	0.53
Zn^2+^	2.34	2.39
Co^2+^	0.075	0.078

As there were not significant variations in the data of both years, therefore, data was pooled together (Additional file 1: Table S1).

### Effect of *A. lipoferum, A. vinelandii* and NP fertilizers on leaf chlorophyll and protein contents, plant height and no. of branches plant^− 1^

**Fig. 1:**
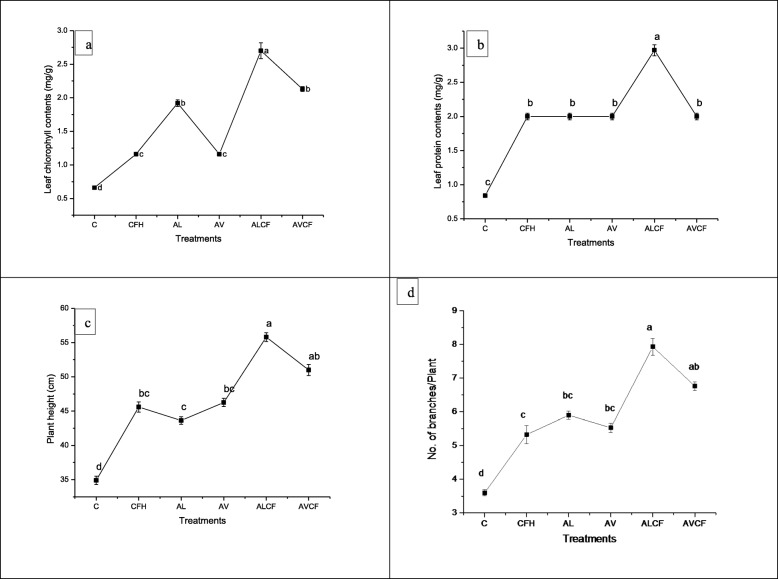
Effect of *Azospirillum lipoferum*, *Azotobacter vinelandii* and NP fertilizers on (**a**) leaf chlorophyll contents, **b** leaf protein contents, **c** plant height and (**d**) no. of branches plant^− 1^ in Sesame. Means with at least one common letter are not significantly different at the *P* < 0.05. LSD values for leaf chlorophyll contents (0.1770); leaf protein contents (0.0927); plant height (2.0643) and no. of branches plant^− 1^ (0.4369). Detail of treatments as described below. C, Control; CFH, half dose of NP fertilizers; AL, *Azospirillum lipoferum*; AV, *Azotobacter vinelandii; ALCF, azospirillum lipoferum* + half dose of NP fertilizers; AVCF, *Azotobacter vinelandii* + half dose of NP fertilizers

All the treatments resulted in increase in leaf chlorophyll and protein contents as compared to the control (Fig. 1a and b). However, maximum increase (309%) in leaf chlorophyll content was recorded in *A. lipoferum* + half dose of NP fertilizer treatment (ALCF) followed by single application of *A. lipoferum* (190%). Maximum leaf soluble protein content (253%) was recorded by ALCF treatment and rest of the treatments showed similar increase of 138% over the control (Fig. 1b).

Significant increase in plant height and number of branches plant^− 1^ was recorded by all the treatments. However, maximum increase (60%) in plant height was resulted by ALCF treatment followed by AVCF treatment (*A. vinelandii* + half dose of chemical fertilizer) as compared to that of control (Fig. 1c). Application of ALCF treatment resulted in maximum improvement (120%) in number of branches plant^− 1^ followed by AVCF treatment (Fig. 1d). Other treatments also showed substantial increase over the control.

### Effect of *A. lipoferum, A. vinelandii* and NP fertilizers on number of capsule branch^− 1^, seed yield and seed oil contents

**Fig. 2:**
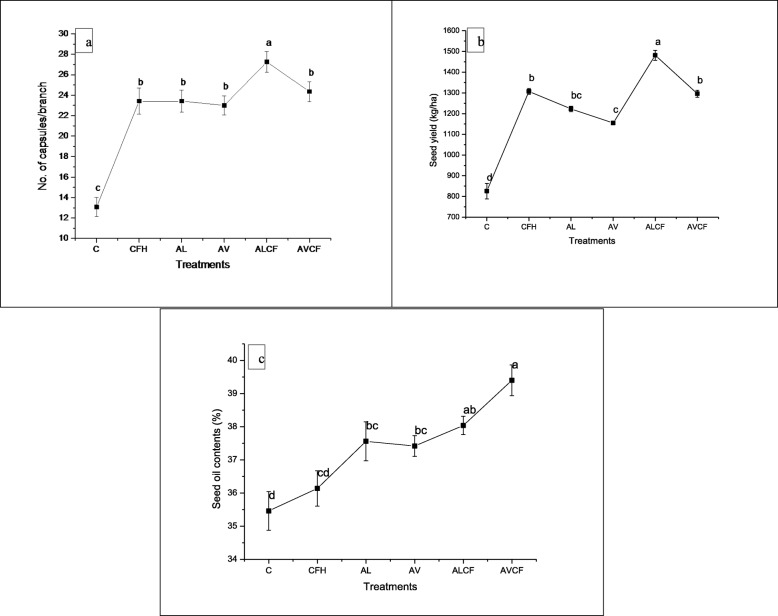
Effect of *Azospirillum lipoferum*, *Azotobacter vinelandii* and NP fertilizers on (**a**) no. of capsules branch^− 1^, **b** seed yield and (**c**) seed oil contents in Sesame. Means with at least one common letter are not significantly different at the *P* < 0.05. LSD values for no. of capsules branch^− 1^ (2.3678); seed yield (54.242) and seed oil contents (1.7676). Detail of treatments as described below. C, Control; CFH, half dose of NP fertilizers; AL, *Azospirillum lipoferum*; AV, *Azotobacter vinelandii; ALCF, azospirillum lipoferum* + half dose of NP fertilizers; AVCF, *Azotobacter vinelandii* + half dose of NP fertilizers

Figure 2a showed that application of the different treatments increased the number of capsules branch^− 1^, however, maximum increment (108%) was recorded by ALCF treatment over the control (Fig. 2a). Rest of the treatments also showed considerable amount of increase as compared to the control.

Seed yield and seed oil contents were notably increased by all the treatments as compared to the control (Fig. 2b and c). However, maximum significant increase (79%) in seed yield was resulted by ALCF treatment (Fig. 2b). Maximum seed oil content (39.40%) was recorded in AVCF treatment followed by ALCF treatment (Fig. 2c) as compared to the control. However, the effect of rest of the treatments viz. *A. lipoferum* (AL), *A. vinelandii* (AV) and CFH on seed oil content was significantly higher as compared to the control.

### Effect of *A. lipoferum, A. vinelandii* and NP fertilizers on fatty acids of sesame oil

**Fig. 3:**
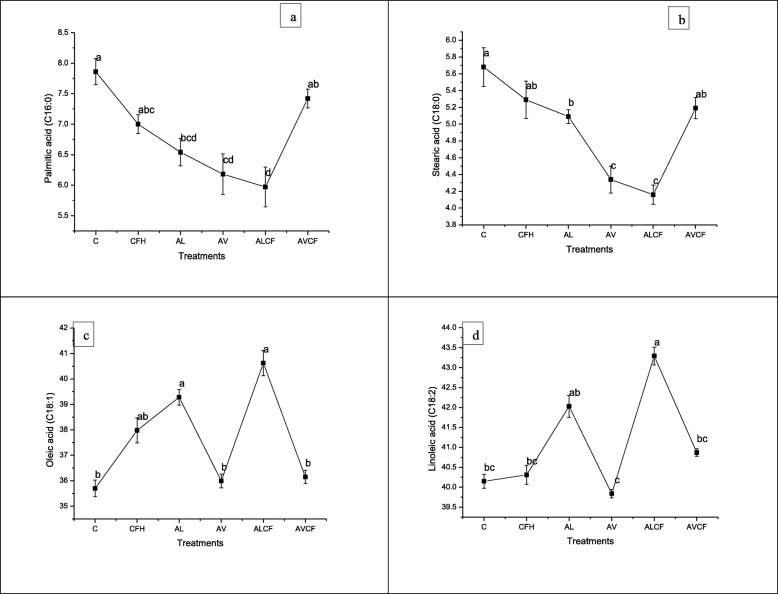
Effect of *Azospirillum lipoferum*, *Azotobacter vinelandii* and NP fertilizers on (**a**) palmitic acid, **b** stearic acid, **c** oleic acid and (**d**) linoleic acid in Sesame. Means with atleast one common letter are not significantly different at the *P* < 0.05. LSD values for palmitic acid (0.3439); stearic acid (0.4526); oleic acid (0.9118) and linoleic acid (0.6060). Detail of treatments as described below. C, Control; CFH, half dose of NP fertilizers; AL, *Azospirillum lipoferum*; AV, *Azotobacter vinelandii; ALCF, azospirillum lipoferum* + half dose of NP fertilizers; AVCF, *Azotobacter vinelandii* + half dose of NP fertilizers.

The results in Fig. 3a-d indicated that treatments ALCF and AVCF were highly effective in altering the relative proportion of major fatty acids of sesame oil. The treatments ALCF exhibited significantly lower palmitic acid (C 16:0) and stearic acid content over the control (Fig. 3a and b). The AVCF treatment showed non-significant difference in palmitic and stearic acid (C18:0) content as compared to the control (Fig. 3a and b).

Maximum increase (6%) in oleic acid contents (C18:1) was recorded by ALCF treatment followed by AL treatment as compared to the control (Fig. 3c). Significantly higher increase (7%) in linoleic acid (C18:2) was also observed in ALCF treatment as compared to the control (Fig. 3d). However, a reduction in linoleic acid was recorded in the treatment *A. vinelandii* (AV) applied without fertilizer.

### Effect of *A. lipoferum, A. vinelandii* and NP fertilizers on iodine value, saponification number acid value and free fatty acid contents

**Fig. 4:**
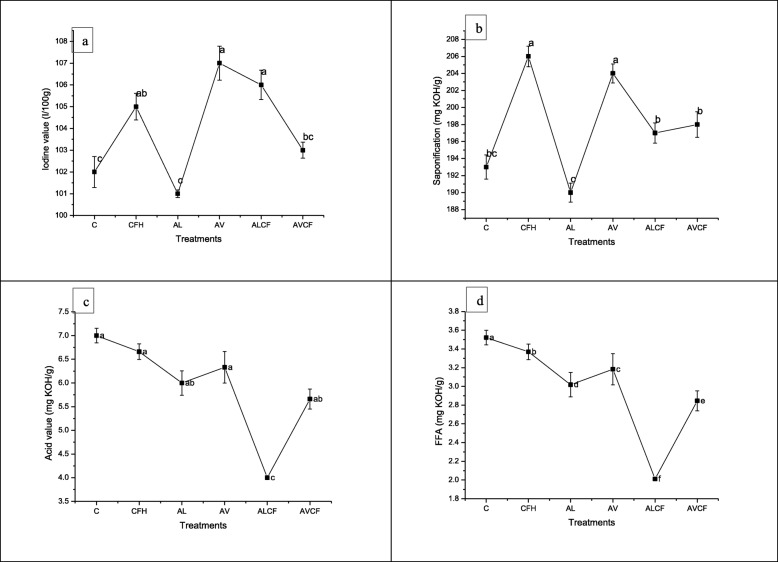
Effect of *Azospirillum lipoferum*, *Azotobacter vinelandii* and NP fertilizers on (**a**) iodine value, **b** saponification number, **c** acid value and (**d**) free fatty acid and in Sesame. Means with at least one common letter are not significantly different at the *P* < 0.05. LSD values for iodine value (4.5327), saponification number (5.1052), acid value (0.5803) and free fatty acid (0.2919). Detail of treatments as described below. C, Control; CFH, half dose of NP fertilizers; AL, *Azospirillum lipoferum*; AV, *Azotobacter vinelandii; ALCF, azospirillum lipoferum* + half dose of NP fertilizers; AVCF, *Azotobacter vinelandii* + half dose of NP fertilizers

Results showed that maximum reduction in iodine value and saponification number was recorded by *A. lipoferum* (AL) when applied alone (Fig. 4a and b), however, this decrease was not significant as compared to the control. Rest of the treatments showed increase in both the parameters over the control. All the treatments showed reduction in acid value, however, maximum significant reduction in acid value (42%) was recorded in the oil of ALCF treatment as compared to the control (Fig. 4c).

Free fatty acid contents were significantly reduced by all the treatments, however, maximum reduction (50 and 29%) was recorded by ALCF followed by AVCF treatments respectively over the control (Fig. 4d). Rest of the treatments also significantly reduced the free fatty acid contents as compared to the control.

## Discussion

**Fig. 5:**
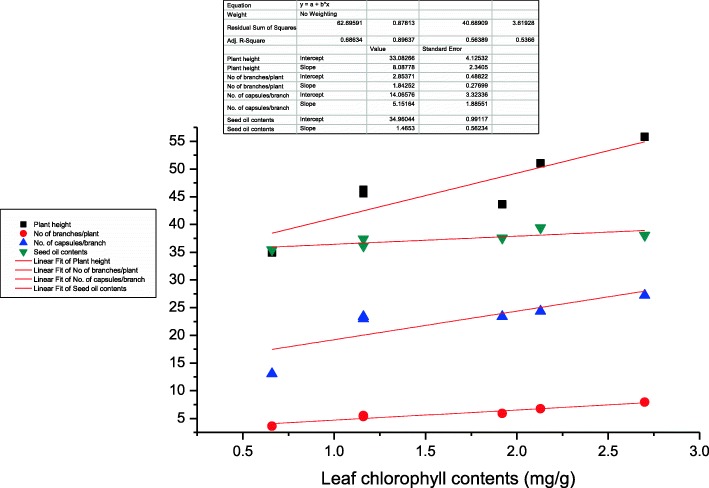
Regression analysis between leaf chlorophyll contents and plant height, no. of branches/plant, no. of capsules, seed oil contents

**Fig. 6:**
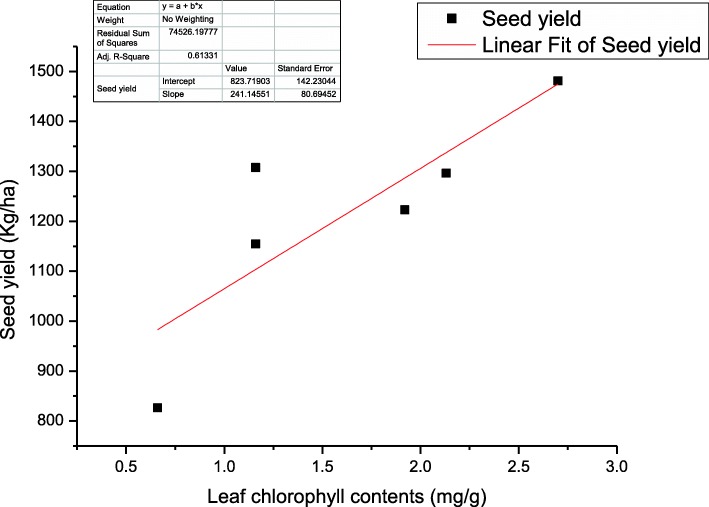
Regression analysis between leaf chlorophyll contents and seed yield

Beneficial effects of *A. lipoferum* inoculation in combination with half dose of NP fertilizers (ALCF) on increased chlorophyll and protein contents might be due to the increased supply of nitrogen to the developing tissue and organs. The nitrogen fertilizers improved the chlorophyll contents of a plant and it was easily observed by the dark green color of the plant [14, 15]. *A. lipoferum* is a nitrogen fixer which can provide the increased amount of nitrogen which might enhanced the production of amino acid and ultimately lead to the increase in protein contents. Shakeri et al. [9] reported that application of nitrogen fertilizers and nitrogen fixing rhizobacteria significantly improved the protein content in sesame. It has been reported that inoculation of oil palm plants with *A. lipoferum* significantly improved the chlorophyll content [16] and these findings are in line with our results except that of tested strains enhanced yield and oil quality along with the half dose of nitrogen as well as phosphorous fertilizer. *Azospirillum* is a N_2_-fixing organism and assist in increasing nitrogen metabolism and leaf protein content. Similar observations were reported by Haroun and Hussein [17] in *Lupinus* seedling that biofertilizers enhanced the relative protein concentration. The increase in plant height was more pronounced in ALCF treatment. Increase in leaf chlorophyll contents leads to increase level of photosynthesis which is correlated with increased amount of carbohydrates production, which ultimately leads to the increase in growth components of the plant in the form of plant height and number of branches per plant and ultimately seed yield (Figs. 5 and 6). There is a positive correlation between leaf chlorophyll contents and other agronomic and seed yield traits (plant height and number of branches plant^− 1^, no. of capsules branch^− 1^) at *P* < 0.001 (Table 2). Similarly, phosphate fertilizers increased the cell division rate and other metabolic activities and leads to increase growth of plant. Similar findings were reported by Chandrasekar et al. [18] that the combined application of biofertilizers and agro-fertilizers significantly improved the plant height in sesame and the seed yield of *Echinochloa frumentacea*. Increase in no. of branches, no. of capsules per branch and seeds per capsule lead towards overall increase in seed yield and a positive correlation is shown in Table 2 at *P* < 0.001. Shakeri et al. [9] reported that application of PGPR increased the yield and yield components in sesame. It was reported in a study that about 70% of total nitrogen requirement of the host plant was supplied by the application of *Azospirillum* and other rhizospheric bacteria [19] which leads to increase in yield of the crop. Likewise, Soleimanzadeh et al. [20] reported that PGPR use the important mechanism of plant growth substances production, which leads towards the increased development and growth of plant. In the current study, maximum increase in seed oil contents was resulted in AVCF and it is according to the previous study that *A. vinelandii* improved the seed oil content as compared to the control and agrochemical [12]. The increase in oil contents leads to the increase in net yield of oil which is an important parameter from improved oil quantity perspectives. Similar findings were reported by Nosheen et al. [12] that the improvement in oil content of seed was recorded in *Azotobacter* treatment in canola which is in corroboration of the current results. *A. vinelandii* alone (AV) and ALCF treatment increased iodine value. However, there must be a limit in increase in this parameter because the increase in iodine up to an optimum limit produced bad quality oil with perspective to edible point of view as it causes the oxidation instability of the oil. The ALCF as well as AVCF were highly effective in altering the fatty acid composition and improving the quality of sesame oil. In the current study, palmitic and stearic acid (saturated fatty acids) were increased by AVCF while ALCF significantly increased the oleic and linoleic acid (unsaturated fatty acids) contents of sesame seed. Shehata and El-Khawas [21] reported that application of biofertilizers on sunflower resulted in alteration in the composition of fatty acid. Similarly, Nosheen et al. [12] reported that inoculation of canola with *Azotobacter* and *Azospirillum* increased the oleic acid and linolenic acid contents which are in agreement of our findings. Sharifi et al. [22] reported that there is a reverse relationship between saturated and unsaturated fatty acid, and the similar trends is shown in our study, the treatments which increased the saturated fatty acid leads toward decrease in unsaturated fatty acid and vice versa. Significant negative correlation (*r* = − 0.4542, *r* = − 0.2818) between the saturated and unsaturated fatty acid has been shown in Table 2. Similarly, it has been evident from a previous study that *Azospirillum brasilense-*ACP-transformation in brassica resulted in prime increased in oleic acid (C18:1) and linoleic acid (C18:2) with parallel decrease in other fatty acids. Acyl carrier protein (ACP) regulates the production of fatty acids in the plant and bacteria [23] which perform specific functions, therefore, regulation of ACP for specific fatty acid might be the reason of increase production of oleic and linoleic acid as compared to palmitic and stearic acid. As we know that oleic acid is an important fatty acid for good quality edible oil production as its increased production is important to create a balance between saturated and unsaturated fatty acid. Moreover, it is also reported that oleic acid might be responsible for the hypotensive (blood pressure reducing) effects of oil [24]. Similarly, Perdomo et al. [25] reported the importance of oleic acid at cardiovascular level. He described that the oleic acid has a beneficial effect as compared to saturated fatty acids at cardiovascular level. Consequently, the improvement in endothelial dysfunction, inflammation, protection against resistance of cardiovascular insulin and reduction in proliferation and apoptosis in VSMCs (vascular smooth muscle cells) that may contribute to an ameliorated atherosclerotic process and its stability are due to oleic acid. The biofertilizers in combination with NP fertilizers decreased the content of saturated fatty acids and hence may be implicated to improve properties of sesame oil. Shakeri et al. [9] reported that application of PGPR and half dose of nitrogen fertilizers decreased the saturated fatty acid and increased the unsaturated fatty acid which are in accordance of our results in which Azospirillum in combination with half dose of NP fertilizers increased unsaturated fatty acid over saturated fatty acid contents as compared to other treatments. Abeer et al. [26] reported that improved root, shoot growth, phospholipid fraction, lipid content, photosynthetic pigments and increased fatty acids contents in *Indian bassia* plant leaves were recorded by the application of *B. subtilis* which are in accordance to the current findings. Similarly, Jha et al. [23] reported that transformation of *Azospirillum lipoferum* ACP and its functional expression in *Brassica juncea* improved the fatty acid profile predominantly C18:1 fatty acid, it shows that *Azospirillum lipoferum* has capability to alter the fatty acid composition and improve the concentration of oleic acid.

**Table 2:** Pearson correlation coefficient for all parameters

Acid Value		No. of Branches /plant	No. of Capsules /branch	Leaf Chlorophyll	Free Fatty Acid	Iodine value	Linoleic acid	Oleic acid	Palmitic acid	Plant height	Leaf Protein	Saponific-ation number	Seed yield	Stearic acid
No. of Branches /plant	−0.7803*** 0.0000													
No. of Capsules /branch	−0.6051*** 0.0001	0.6920*** 0.0000												
Leaf Chlorophyll	−0.8427*** 0.0000	0.8847*** 0.0000	0.7214** 0.0000											
Free Fatty Acid	1.0000*** 0.0000	−0.7803*** 0.0000	−0.6051*** 0.0001	− 0.8427*** 0.0000										
Iodine value	−0.1147 0.5055	0.2639 0.1199	0.0071 0.9671	0.0092 0.9576	−0.1147 0.5055									
Linoleic acid	−0.7466*** 0.0000	0.6521*** 0.0000	0.4618* 0.0046	0.7833*** 0.0000	−0.7466*** 0.0000	−0.0983 0.5686								
Oleic acid	−0.5924*** 0.0001	0.5974*** 0.0001	0.4755* 0.0034	0.6378*** 0.0000	−0.5924*** 0.0001	−0.0023 0.9893	0.7581*** 0.0000							
Palmitic acid	0.5678** 0.0003	−0.4336* 0.0082	−0.4468* 0.0063	− 0.4229* 0.0102	0.5678*** 0.0003	− 0.2408 0.1572	−0.4281* 0.0092	− 0.4542 − 0.4542						
Plant height	−0.7904*** 0.0000	0.9037*** 0.0000	0.7813*** 0.0000	0.8362*** 0.0000	−0.7904*** 0.0000	0.2353 0.1671	0.5069*** 0.0016	0.4730* 0.0036	−0.4890* 0.0025					
Leaf Protein	−0.7637*** 0.0000	0.8820*** 0.0000	0.8497*** 0.0000	0.8202*** 0.0000	−0.7637*** 0.0000	0.1978 0.2474	0.6587*** 0.0000	0.7158*** 0.0000	−0.5843*** 0.0002	0.8935*** 0.0000				
Saponification number	0.1049 0.5428	0.1066 0.5362	0.1641 0.3389	−0.1626 0.3435	0.1049 0.5428	0.1942 0.2564	−0.2936 0.0822	−0.0707 0.6818	0.0182 0.9160	0.1889 0.2698	0.2122 0.2141			
Seed yield	−0.7118*** 0.0000	0.8523*** 0.0000	0.8381*** 0.0000	0.7822*** 0.0000	−0.7118*** 0.0000	0.1812 0.2903	0.6058*** 0.0001	0.6156*** 0.0001	−0.4778* 0.0032	0.8772*** 0.0000	0.9237*** 0.0000	0.1852 0.2795		
Stearic acid	0.5250*** 0.0010	−0.5722*** 0.0003	−0.5272*** 0.0010	− 0.4574*** 0.0050	0.5250*** 0.0010	− 0.2701 0.1111	−0.3272* 0.0514	− 0.2818 0.0958	0.4918* 0.0023	− 0.5570*** 0.0004	−0.6273*** 0.0000	− 0.2404 0.1579	−0.4719* 0.0037	
Seed oil contents	−0.3200* 0.0571	0.4812* 0.0030	0.4858* 0.0027	0.4912* 0.0023	−0.3200* 0.0571	−0.3748 0.0243	0.2644 0.1191	0.1685 0.3258	0.0393 0.8198	0.4435* 0.0067	0.4231 0.0101	0.2093 0.2206	0.4249* 0.0098	−0.2407 0.1573

Means labelled with *** showed a significant correlation at *P* ≤ 0.001, with ** at *P* ≤ 0.01, and with * at *P* ≤ 0.05

Increased acid value and free fatty acid contents cause oxidation of oil. Lower acid values are favorable for good quality edible oil production. In the current study, *Azospirillum* with half dose of NP fertilizers significantly lowered the acid value as compared to the control and other treatments, however, the mechanism of alteration in oil quality by PGPR is not completely explored yet. These results are in parallel to that of Abd El-Gawad et al. [27] who reported that *Azotobacter chroococcum and Bacillus megaterium* reduced the acid value and peroxide value of canola when compared to that of untreated control. Decrease in acid value might be due to the production of phytohormone by PGPR as Ullah et al. [28] reported that application of cytokinin decreased the acid value of safflower oil. Omer and Abd-Elnaby [29] reported that application of biofertilizers in interaction with foliar application of KCl improved the yield, seed oil contents and seed chemical constituents of sesame. The rhizobacteria strains *Azospirilum* and *Azotobacter* are ideal candidate for development of bioformulation for the sesamum plant. However, their compatibility and synergistic efficacy with other microbes like AM fungi need to be tested under diverse ecological conditions.

## Conclusion

The treatment ALCF was highly effective in improving yield and quality of sesame oil in term of increase in oleic acid contents and other quality traits as compared to other treatments which are important findings of the study. Furthermore, the application of ALCF and AVCF also reduced the use of NP fertilizers to about 30–50% which was the aim of the study. Therefore, it can be concluded that application of ALCF treatment not only reduced the amount of chemical fertilizer but also improved growth, yield and quality of sesame oil with perspective to edible oil production and can be suggested as best treatment. However, further molecular approaches are needed to confirm these finding and to explore the mechanism which are involved in altering the physiology and fatty acid composition of sesame.

## Methods

The field experiments were conducted at the fields of Quaid-i-Azam University Islamabad during the months of July to October of two consecutive years 2012–2013. Weather data of both the years (2012 and 2013) has been given in Additional file 2: Table S2.

Field plots measuring 3 × 3 m^2^ comprised of three replications for each treatment arranged in a randomized complete block design (RCBD). Seeds of sesame (*Sesamum indicum* L.) cultivar TS-3, were obtained from NARC (National Agricultural Research Center) Islamabad. Seeds were sterilized with 95% ethanol and 0.1% mercuric chloride (HgCl_2_), afterwards washed with sterile water.

The PGPR strains *A. lipoferum* (Accession no.GQ255949) was isolated from the rhizosphere of maize and *A. vinelandii* Khsr1 (Accession no. GQ849485) was screened from the root of *Chrysopogon aucheri* in the previous study [30].

### Treatments

Following treatments were used: C: control, CFH: (Half dose of the recommended chemical fertilizers (urea 25 kg/ha + Diammonium Phosphate 30 kg/ha), AL: (*Azospirillum lipoferum*), AV: (*Azotobacter vinelandii), ALCF (azospirillum lipoferum* + urea (25 kg/ha as half dose) + Diammonium Phosphate (30 kg/ha as half dose), AVCF (*Azotobacter vinelandii* + urea (25 kg/ha as half dose) + Diammonium Phosphate (30 kg/ha as half dose).

The *A. lipoferum* and *A. vinelandii* were applied (10^8^ cfu/mL) as seed inoculation [31]. For the preparation of inoculum, Luria Bertani media (LB) was used for the inoculation of both bacterial strains (24 h old cultures) in order to prepare the broth culture. After shaking at 24 °C for 72 h, the centrifugation was carried out for 10 min at 10,000 rpm and pellet was collected. The supernatant was castoff and pellet were diluted up to 100 mL with autoclaved water in order to get the optical density one at 600 nm. Already sterilized seed were then soaked in the bacterial culture for 6 hours and sowing was carried out. Cell free supernatant was used in control treatment.

Application of fertilizers as half dose of DAP was carried out at the time of sowing while half dose of urea was applied at an interval of 40 days into three equal parts. The irrigations were applied as required.

Sampling of leaf for the chlorophyll and protein contents was carried out during vegetative stage of the plant. However, the agronomic data (plant height, number of branches plant^− 1^, number of capsule branch^− 1^, and seed yield) was collected at crop maturity. About 10–15 plants per replications were sampled and observed for each parameter except the yield, for which whole plants were harvested, crushed and weighed.

### Determination of leaf chlorophyll content

Leaf chlorophyll content was determined according to the method of Hiscox and Israelstam [32] using Dimethyl Sulfoxide (DMSO). Small leaf discs were extracted in DMSO at 65 °C until the leaf discs became completely colourless. Absorbance of chlorophyll extracted in DMSO was measured at 665 and 645 nm with spectrophotometer (Hitachi U-1500).

### Determination of leaf soluble protein content

Protein content of the leaves was determined following the method of Lowry et al. [33]. Grinding of fresh leaves (0.1 g) were made in phosphate buffer of 7.5 pH (1 mL) using a pestle and mortar. Ground samples were centrifuged at 3000 rpm at 4 °C for 10 min and supernatant (0.1 mL) was collected. Alkaline copper sulphate reagent was added to supernatant in 1:1 ratio and was shaken for 10 min before adding Folin’s reagent (0.1 mL). After 30 min, the absorbance was recorded at 650 nm for each sample. The concentration of protein was calculated by standard curve made from different concentrations of Bovin Serum Albumin (BSA) as a reference.

### Estimation of seed oil content

Seed oil content was estimated by Nuclear Magnetic Resonance spectroscopy (NMR, Oxford Analytical, UK, New Port 4000) according to Robertson and Morrison [34]. Nuclear magnetic resonance spectroscopy is a non-destructive method for the determination of seed oil contents which is based on the absorption of energy by an atomic nucleus changing its spin orientation in the magnetic field.

### Oil extraction

Oil from sesame seed was extracted using n-hexane as solvent at 60 °C for 6 h with the help of Soxhlet apparatus according to the AOCS Ag 1–65 method [35].

### Fatty acids analysis

For quantification of fatty acids, the AOCS standard method Ce 2–66 [36] was used for the preparation of fatty acid methyl esters (FAME). The analyses of FAME (0.5 mL) was carried out in a gas chromatograph (Shimadzu QP 5050) having a fused silica capillary column (MN FFAP (50 m _ 0.32 mm i.d. film thickness 0.25 mm) and flame ionizing detector (FID). The gas used as a carrier was Helium and the temperature of the column was maintained at 110 °C for 0.5 min and then heated to 200 °C at 10 °C/min, then maintained for 10 min. Injector and detector temperatures were set at 220 °C and 250 °C, respectively [37].

### Estimation of iodine value

Estimation of iodine value of sesame oil was carried out according to the method recommended by AOAC [38]. Oil sample (0.2 g) was taken in 100 mL glass stoppered bottle and dissolved in 15 mL of carbon tetrachloride solution. After addition of 25 mL Wij’s reagent the contents, the flask was kept on standing at 25 °C for 2 h in dark and then 20 mL of potassium iodide (10% w/v) was added and titrated with sodium thiosulphate (0.2 N) using starch as indicator. A blank was prepared in the similar manner and iodine value was calculated.

### Determination of acid value

Acid value of sesame oil was determined using the method of Cox and Pearson [39]. Oil sample (0.2 g) was dissolved in 2.5 mL of ethanol:diethylether in the ratio of 1:1 and titrated with NaOH [0.1 N]. Phenolphthalein was used as indicator and acid value was calculated.

### Determination of free fatty acid

Free fatty acid of sesame oil samples was calculated by multiplying the acid value with 0.503 factor. The formula is given as % Free Fatty Acid = 0.503 × acid value.

### Determination of saponification value

Saponification value was estimated according to the method of Pearson [40]. Oil sample (1 g) was taken in a flask and 12.5 mL of ethanolic potassium hydroxide was added and then heated for 30 min in boiling water and titrated against 0.5 N hydrochloric acid (HCl) using phenolphthalein (1% v/v) as indicator. A blank determination was also accomplished under similar condition and saponification value was calculated.

### Determination of specific gravity

Specific gravity was determined according to the method of AOAC [41].

### Statistical analysis

Data was analyzed by Analysis of Variance (ANOVA) using Statistix software version 8.1 and mean values were compared by least significant difference (LSD) at *P* < 0.05 according to Steel and Torrie [42]. Graphical representation of the data was carried out by OriginPro 2016 (OriginLab, Northampton, MA).

## Supplementary information

**Additional file 1: Table S1.** Analysis of variance (ANOVA).

**Additional file 2: Table S2.** Weather data of both years (2012 and 2013) of the experiment.

## Data Availability

All data generated or analysed during this study are included in this published article [and its supplementary information files are given as Additional file 1: Table S1 and Additional file 2: Table S2.

## Abbreviations

ACP: Acyl carrier protein
AL: *Azospirillum lipoferum*
ALCF: *Azospirillum lipoferum* + Half dose of chemical fertilizers
ANOVA: Analysis of Variance
AOAC: Association of Official Analytical Chemists
AOCS: American oil chemist’s society
AV: *Azotobacter vinelandii*
AVCF: *Azotobacter vinelandii* + Half dose of chemical fertilizers
BSA: Bovin serum albumin
C: Control
CFH: Half dose of chemical fertilizers
DAP: Diammonium Phosphate
DMSO: Dimethyl Sulfoxide
FAMES: Fatty acid methyl esters
*FFAP*: Free fatty acid phase
FID: Flam ionization detector
g: gram
HCl: Hydrochloric acid
LSD: Least significant difference
mL: milliliter
N: Nitrogen
NaOH: Sodium hydroxide
NARC: National Agricultural Research Center
NMR: Nuclear magnetic resonance spectroscopy
NP: Nitrogen phosphorus
PGPR: Plant Growth Promoting Rhizobacteria
RCBD: Randomized complete block design
VSMCs: Vascular smooth muscle cells

## References

[1] Barcelos E, de Almeida RS, Cunha RNV, Lopes R, Motoike SY, Babiychuk E, Skirycz A, Kushnir S (2015). Oil palm natural diversity and the potential for yield improvement. Front Plant Sci.

[2] Dossa K, Wei X, Li D, Fonceka D, Zhang Y, Wang L, Yu J, Boshou L, Diouf D, Cissé N, Zhang X (2016). Insight into the AP2/ERF transcription factor superfamily in sesame and expression profiling of DREB subfamily under drought stress. BMC Plant Biol.

[3] Elkhaleefa A, Shigidi I (2015). Optimization of sesame oil extraction process conditions. Adv Chem Eng Sci.

[4] Mirzakhani M, Ardakani MR, Aeene Band A, Shirani Rad AH, Rejali F. Effects of dual inoculation of Azotobacter and Mycorrhiza with nitrogen and phosphorus fertilizer rates on grain yield and some of characteristics of spring safflower. Int J Civil Environ Eng. 2009;1:39-43.

[5] Saeed KS, Ahmed SA, Hassan IA, Ahmed PH (2015). Effect of bio-fertilizer and chemical fertilizer on growth and yield in cucumber (*Cucumis sativus*) in green house condition. Pak J Biol Sci.

[6] Chandini, Kumar R, Kumar R, Prakash O (2019). The Impact of Chemical Fertilizers on our Environment and Ecosystem. Research Trends in Environmental Sciences.

[7] Delaplace P, Delory BM, Baudson C, Cazenave MM, De Spaepen S, Varin S, Brostaux Y, du Jardin P (2015). Influence of rhizobacterial volatiles on the root system architecture and the production and allocation of biomass in the model grass *Brachypodium distachyon* (L.) P. Beauv. BMC Plant Biol.

[8] Porcel R, Zamarreño ÁM, García-Mina JM, Aroca R (2014). Involvement of plant endogenous ABA in *Bacillus megaterium* PGPR activity in tomato plants. BMC Plant Biol.

[9] Shakeri E, Modarres-Sanavy SAM, Dehaghi MA, Tabatabaei SA, Moradi-Ghahderijani M (2016). Improvement of yield, yield components and oil quality in sesame (*Sesamum indicum* L.) by N-fixing bacteria fertilizers and urea. Arch Agron Soil Sci.

[10] El-Habbasha SF, Abd El Salam MS, Kabesh MO (2007). Response of two sesame varieties (*Sesamum indicum L.*) to partial replacement of chemical fertilizers by bio-organic fertilizers. Res J Agri Biol Sci.

[11] Kumar B, Pandey P, Maheshwari DK (2009). Reduction in dose of chemical fertilizers and growth enhancement of sesame (*Sesamum indicum* L.) with application of rhizospheric competent *Pseudomonas aeruginosa* LES4. Eur J Soil Biol.

[12] Nosheen A, Bano A, Ullah F (2011). Nutritive value of canola (*Brassica napus* L.) as affected by plant growth promoting rhizobacteria. Eur J Lipid Sci Technol.

[13] Jahan M, Nassiri Mahallati M, Amiri MB, Ehyay HR (2013). Radiation absorption and use efficiency of sesame as affected by biofertilizers inoculation in a low input cropping system. Indus Crop Product.

[14] Haider S, Kanwal S, Uddin F, Azmat R (2006). Phytotoxicity of Pb: II. Changes in chlorophyll absorption Spectrum due to toxic metal Pb stress on *Phaseolus mungo* and *Lens culinaris*. Pak J Biol Sci.

[15] Shibghatallah MAH, Khotimah SN, Suhandono S, Viridi S, Kesuma T (2013). Measuring leaf chlorophyll concentration from its color: A way in monitoring environment change to plantations. AIP Conference Proceedings.

[16] Azlin CO, Amir HG, Keng CL, Zamzuri I (2007). Effect of growth promoting Rhizobacteria on root formation and growth tissue of cultured oil palm (*Elaeis guineensis* Jacq). Biotechnol.

[17] Haroun SA, Hussein MH (2003). The promotive effect of algal biofertilizers on growth, protein pattern and some metabolic activities of *Lupinus termis* plants grown in siliceous soil. Asian J Plant Sci.

[18] Chandrasekar BR, Ambrose G, Jayabalan N (2005). Influence of biofertilizers and nitrogen source level on the growth and yield of *Echinochloa frumentaceae* (Roxb.) link. J Agric Sci Technol.

[19] Malik KA, Rakhshanda B, Mehnaz S, Rasul G, Mirza MS, Ali S (1997). Association of nitrogen-fixing plant-growth promoting rhizobacteria (PGPR) with kallar grass and rice. Plant Soil.

[20] Soliman AH, Mahmoud AA, Gendy ASH (2012). Effect of foliar fertilizers on growth, yield and active ingredients of safflower plant under sandy soil conditions. J Appl Sci Res.

[21] Shehata MM, El-Khawas SA (2003). Effect of two biofertilizers on growth parameters, yield characters, nitrogenous components, nucleic acids contents, minerals, oil contents, protein profiles and DNA banding pattern of sunflower (*Helianthus annus* L. cv. Vedock) yield. Pak J Biol Sci.

[22] Sharifi RS, Namvar A, Sharifi RS (2017). Grain filling and fatty acid composition of safflower fertilized with integrated nitrogen fertilizer and biofertilizers. Pesq Agropec Bras Brasília.

[23] Jha JK, Sinha S, Maiti MK, Basu A, Mukhopadhyay UK, Sen SK (2007). Functional expression of an acyl carrier protein (ACP) from *Azospirillum brasilense* alters fatty acid profiles in *Escherichia coli* and *Brassica juncea*. Plant Physiol Biochem.

[24] Teres S, Barcelo-Coblijn G, Benet M, Alvarez R, Bressani R, Halver JE, Escriba PV (2008). Oleic acid content is responsible for the reduction in blood pressure induced by olive oil. Proc Natl Acad Sci U S A.

[25] Perdomo L, Beneit N, Otero YF, Escribano Ó, Díaz-Castroverde S, Gómez-Hernández A, Benito M (2015). Protective role of oleic acid against cardiovascular insulin resistance and in the early and late cellular atherosclerotic process. Cardiovas Diabetol.

[26] Abeer H, Abdallah EF, Alqarawi AA, AL-Huqail Asma A, Alshalawi SRM, Wirth S, Dilfuza E (2015). Impact of plant growth promoting *Bacillus subtilis* on growth and physiological parameters of *Bassia indica* (*Indian bassia*) grown udder salt stress. Pak J Bot.

[27] Abd El-Gawad AM, Hendawey MH, Farag HIA (2009). Interaction between biofertilization and canola genotypes in relation to some biochemical constituents under Siwa Oasis conditions. Res J Agric Biol Sci.

[28] Ullah F, Bano A (2011). Effect of plant growth regulators on oil yield and biodiesel production of safflower (*Carthamus tinctorius* L.). Brazilian J Plant Physiol.

[29] Omer Amal, Abd-Elnaby Ahmed (2017). Effect of Phosphate Dissolving Bacteria on Physiological Behavior of Some Sesame Cultivars under Saline Conditions at Sahle Eltina- North Sinai. Alexandria Science Exchange Journal: An International Quarterly Journal of Science Agricultural Environments.

[30] Naz I, Bano A, Rehman B, Pervaiz S, Iqbal M, Sarwar A, Yasmin F (2012). Potential of *Azotobacter vinelandii* Khsr1 as bio-inoculant. African J Biotech.

[31] Noumavo PA, Kochoni GME, Didagbé Y, Adjanohoun A, Allagbe M, Sikirou R, Gachomo E, Kotchoni S, Baba-Moussa L (2013). Effect of different plant growth promoting Rhizobacteria on maize seed germination and seedling development. Amer J Plant Sci.

[32] Hiscox JD, Israelstam GF (1979). A method for the extraction of chlorophyll from leaf tissue without maceration. Can J Bot.

[33] Lowry OH, Poesenbrough NJ, Fal AL, Randall RJ (1951). Protein measurement with folin phenol reagent. J Biol Chem.

[34] Robertson JA, Morrison WH (1979). Analysis of oil content of sunflower seed by NMR. J Amer Oil Chem Soc.

[35] AOAC (2000). Official methods of analysis.

[36] AOCS (1993). Official methods and recommended practices of the American oil chemists’ society.

[37] Camas N, Cırak C, Esendal E (2007). Seed yield, oil content and fatty acids composition of safflower (*Carthamus tinctorius* L.) grown in northern Turkey conditions. J Fac Agric Univ Ondokuz Mayıs.

[38] AOAC (1984). Official methods of Analysis.

[39] Cox HE, Pearson D (1962). The chemical analysis of foods.

[40] Pearson D. Chemical Analysis of Foods. 7th Edn. London: Church Hill Livingstone; 1976. p. 488–96.

[41] AOAC (1970). Official method of analysis.

[42] Steel RGD, Torrie GH (1980). Principles and procedures of statistics.

