# Supplementary material for: Nutritional value of Sesamum indicum L. was improved by Azospirillum and Azotobacter under low input of NP fertilizers
Source: BMC Plant Biol. 2019 Nov 4;19:466. doi: 10.1186/s12870-019-2077-3 (PMC6829804; doi:10.1186/s12870-019-2077-3)
Supplement: Supplementary file 1 — Additional file 1: Table S1. Analysis of variance (ANOVA). [file 12870_2019_2077_MOESM1_ESM.docx]

**Table S1** Analysis of variance (ANOVA).

| **Variables** | **Source** | **DF** | **SS** | **MS** | **F** | **P Value** |
| --- | --- | --- | --- | --- | --- | --- |
| **Leaf chlorophyll contents** | Replicates | 2 | 0.0411 | 0.02055 |  |  |
|  | Treatments | 5 | 16.6691 | 3.33382 | 152.54 | 0.0000 |
|  | Year | 1 | 0.0002 | 0.00023 | 0.01 | 0.9201 |
|  | Treatments ×Year | 5 | 0.0182 | 0.00363 | 0.17 | 0.9724 |
|  | Error | 22 | 0.4808 | 0.02186 |  |  |
|  | Total | 35 | 17.2094 |  |  |  |
| **Leaf protein contents** | Replicates | 2 | 0.1045 | 0.05227 |  |  |
|  | Treatments | 5 | 13.6855 | 2.73709 | 456.20 | 0.0000 |
|  | Year | 1 | 0.1936 | 0.19360 | 32.27 | 0.0000 |
|  | Treatments ×Year | 5 | 0.0480 | 0.00961 | 1.60 | 0.2013 |
|  | Error | 22 | 0.1320 | 0.00600 |  |  |
|  | Total | 35 | 14.1636 |  |  |  |
| **Plant height** | Replicates | 2 | 2.63 | 1.314 |  |  |
|  | Treatments | 5 | 1509.28 | 301.857 | 101.56 | 0.000 |
|  | Year | 1 | 0.42 | 0.422 | 0.14 | 0.7098 |
|  | Treatments ×Year | 5 | 12 | 2.399 | 0.81 | 0.5569 |
|  | Error | 22 | 65.39 | 2.972 |  |  |
|  | Total | 35 | 1589.72 |  |  |  |
| **Number of branches/plant** | Replicates | 2 | 2.4172 | 1.2086 |  |  |
|  | Treatments | 5 | 63.4289 | 12.6858 | 95.27 | 0 |
|  | Year | 1 | 0.0278 | 0.0278 | 0.21 | 0.6523 |
|  | Treatments ×Year | 5 | 0.3422 | 0.0684 | 0.51 | 0.7628 |
|  | Error | 22 | 2.9294 | 0.1332 |  |  |
|  | Total | 35 | 69.1456 |  |  |  |
| **Number of capsules/plant** | Replicates | 2 | 98.842 | 49.421 |  |  |
|  | Treatments | 5 | 694.779 | 138.956 | 35.53 | 0 |
|  | Year | 1 | 9.404 | 9.404 | 2.4 | 0.1352 |
|  | Treatments ×Year | 5 | 2.872 | 0.574 | 0.15 | 0.9789 |
|  | Error | 22 | 86.031 | 3.911 |  |  |
|  | Total | 35 | 891.929 |  |  |  |
| **Seed yield** | Replicates | 2 | 20705 | 10352 |  |  |
|  | Treatments | 5 | 1447137 | 289427 | 141.03 | 0 |
|  | Year | 1 | 13034 | 13034 | 6.35 | 0.0195 |
|  | Treatments ×Year | 5 | 2260 | 452 | 0.22 | 0.95 |
|  | Error | 22 | 45149 | 2052 |  |  |
|  | Total | 35 | 1528285 |  |  |  |
| **Seed oil contents** | Replicates | 2 | 51.389 | 25.6944 |  |  |
|  | Treatments | 5 | 60.806 | 12.1611 | 5.58 | 0.0018 |
|  | Year | 1 | 1.361 | 1.3611 | 0.62 | 0.4378 |
|  | Treatments ×Year | 5 | 2.806 | 0.5611 | 0.26 | 0.9314 |
|  | Error | 22 | 47.944 | 2.1793 |  |  |
|  | Total | 35 | 164.306 |  |  |  |
| **Palmitic acid** | Replicates | 2 | 1.7222 | 0.86111 |  |  |
|  | Treatments | 5 | 14.3056 | 2.86111 | 11.56 | 0 |
|  | Year | 1 | 2.7778 | 2.77778 | 11.22 | 0.0029 |
|  | Treatments ×Year | 5 | 0.8889 | 0.17778 | 0.72 | 0.6165 |
|  | Error | 22 | 5.4444 | 0.24747 |  |  |
|  | Total | 35 | 25.1389 |  |  |  |
| **Stearic acid** | Replicates | 2 | 0.5972 | 0.29861 |  |  |
|  | Treatments | 5 | 9.6847 | 1.93694 | 13.56 | 0 |
|  | Year | 1 | 0.2336 | 0.23361 | 1.64 | 0.2143 |
|  | Treatments ×Year | 5 | 0.9314 | 0.18628 | 1.3 | 0.2984 |
|  | Error | 22 | 3.1428 | 0.14285 |  |  |
|  | Total | 35 | 14.5897 |  |  |  |
| **Oleic acid** | Replicates | 2 | 3.236 | 1.6178 |  |  |
|  | Treatments | 5 | 125.351 | 25.0703 | 43.23 | 0 |
|  | Year | 1 | 5.214 | 5.2136 | 8.99 | 0.0066 |
|  | Treatments ×Year | 5 | 3.585 | 0.7169 | 1.24 | 0.3259 |
|  | Error | 22 | 12.758 | 0.5799 |  |  |
|  | Total | 35 | 150.143 |  |  |  |
| **Linoleic acid** | Replicates | 2 | 0.0572 | 0.0286 |  |  |
|  | Treatments | 5 | 50.6056 | 10.1211 | 39.51 | 0 |
|  | Year | 1 | 0.01 | 0.01 | 0.04 | 0.8452 |
|  | Treatments ×Year | 5 | 1.28 | 0.256 | 1 | 0.4412 |
|  | Error | 22 | 5.6361 | 0.2562 |  |  |
|  | Total | 35 | 57.5889 |  |  |  |
| **Iodine value** | Replicates | 2 | 300.722 | 150.361 |  |  |
|  | Treatments | 5 | 176.222 | 35.244 | 2.46 | 0.0648 |
|  | Year | 1 | 0.111 | 0.111 | 0.01 | 0.9306 |
|  | Treatments ×Year | 5 | 53.556 | 10.711 | 0.75 | 0.5966 |
|  | Error | 22 | 315.278 | 14.331 |  |  |
|  | Total | 35 | 845.889 |  |  |  |
| **Saponification number** | Replicates | 2 | 545.39 | 272.694 |  |  |
|  | Treatments | 5 | 1195.47 | 239.094 | 13.15 | 0 |
|  | Year | 1 | 1.36 | 1.361 | 0.07 | 0.7869 |
|  | Treatments ×Year | 5 | 76.81 | 15.361 | 0.84 | 0.5326 |
|  | Error | 22 | 399.94 | 18.179 |  |  |
|  | Total | 35 | 2218.97 |  |  |  |
| **Acid value** | Replicates | 2 | 1.5 | 0.75 |  |  |
|  | Treatments | 5 | 34.9792 | 6.99583 | 29.79 | 0 |
|  | Year | 1 | 0.0625 | 0.0625 | 0.27 | 0.6111 |
|  | Treatments ×Year | 5 | 1.4792 | 0.29583 | 1.26 | 0.3162 |
|  | Error | 22 | 5.1667 | 0.23485 |  |  |
|  | Total | 35 | 43.1875 |  |  |  |
| **Free fatty acid contents** | Replicates | 2 | 0.3795 | 0.18976 |  |  |
|  | Treatments | 5 | 8.85 | 1.77001 | 29.79 | 0 |
|  | Year | 1 | 0.0158 | 0.01581 | 0.27 | 0.6111 |
|  | Treatments ×Year | 5 | 0.3742 | 0.07485 | 1.26 | 0.3162 |
|  | Error | 22 | 1.3072 | 0.05942 |  |  |
|  | Total | 35 | 10.9268 |  |  |  |

**DF:** degree of freedom

**SS:** Sum of Squares

**MS:** mean square

**F:**  F-statistic
