# Supplementary material for: Nutritional value of Sesamum indicum L. was improved by Azospirillum and Azotobacter under low input of NP fertilizers
Source: BMC Plant Biol. 2019 Nov 4;19:466. doi: 10.1186/s12870-019-2077-3 (PMC6829804; doi:10.1186/s12870-019-2077-3)
Supplement: Supplementary file 2 — Additional file 2: Table S2. Weather data of both years (2012 and 2013) of the experiment. [file 12870_2019_2077_MOESM2_ESM.docx]

**Table S2** Weather data of both years (2012 and 2013) of the experiment.

| **2012** | | | | | | | | |
| --- | --- | --- | --- | --- | --- | --- | --- | --- |
| **Date** | **Max Temp (C^o^)** | **Min  Temp (C^o^)** | **Wind Speed. km/day** | **Pan Evap(mm)** | **Rainfall (mm)** | **Relative Humidity (%)** | **Relative Humidity (%)** | **Avg. Relative Humidity (%)** |
|  |  |  |  |  |  |  |  |  |
| **Jan** | 17.2580 | 2.451613 | 13.61065 | 1.24870968 | 17.25 | 93.1612903 | 65.322581 | 79.241935 |
| **Feb** | 17.3928 | 6.464286 | 36.81107 | 1.48538462 | 9.8 | 94.0357143 | 67.178571 | 80.607143 |
| **March** | 25.2580 | 10.54839 | 22.48613 | 2.71741935 | 2.769677 | 89.1935484 | 58.903226 | 74.048387 |
| **April** | 29.1666 | 14.53333 | 38.08933 | 4.44933333 | 1.202333 | 75.4333333 | 52.133333 | 63.783333 |
| **May** | 36.6129 | 18.46774 | 54.61387 | 7.43225806 | 0.714516 | 58.2580645 | 33.83871 | 46.048387 |
| **June** | 37.8 | 22.9 | 79.52733 | 7.121 | 3.429333 | 68.5 | 48.533333 | 58.516667 |
| **July** | 34.2580 | 23.70968 | 61.12161 | 4.95333333 | 9.357 | 88.4193548 | 69.967742 | 79.193548 |
| **Aug** | 31.6612 | 22.82258 | 39.43516 | 3.69888889 | 17.99806 | 93.3870968 | 77.129032 | 85.258065 |
| **Sep** | 32.1333 | 21.06667 | 38.33133 | 3.85533333 | 4.986 | 89.3333333 | 69.6 | 79.466667 |
| **Oct** | 29.5161 | 16.58065 | 37.32097 | 2.95903226 | 0.406129 | 91.3225806 | 65.258065 | 78.290323 |
| **Nov** | 23.6333 | 6.766667 | 28.60733 | 1.855 | 0.619 | 92.5666667 | 50.033333 | 71.3 |
| **Dec** | 19.5483 | 4.016129 | 25.65484 | 1.31354839 | 0.071613 | 89.8709677 | 49.451613 | 69.66129 |
| **2013** | | | | | | | | |
| **Date** | **Max Temp (C^o^)** | **Min  Temp (C^o^)** | **Wind Speed km/day** | **Pan Evap (mm)** | **Rainfall (mm)** | **Relative Humidity (%)** | **Relative Humidity (%)** | **Avg. Relative Humidity (%)** |
|  |  |  |  |  |  |  |  |  |
| **Jan** | 16.9 | 1.32 | 32.6 | 1.35 |  | 89 | 48 | 68 |
| **Feb** | 17.4 | 3.24 | 20.5 | 1.77 | 1.58 | 88 | 51 | 70 |
| **March** | 24.9 | 8.9 | 23.8 | 3.32 | 0.51 | 79 | 38 | 59 |
| **April** | 29.9 | 15.0 | 34.1 | 4.70 | 1.36 | 66 | 43 | 55 |
| **May** | 36.2 | 18.3 | 69.7 | 7.34 | 0.31 | 51 | 26 | 38 |
| **June** | 40.9 | 22.7 | 80.2 | 10.36 | 0.15 | 41 | 21 | 31 |
| **July** | 35.9 | 31.5 | 68.6 | 6.7 | 6.4 | 74 | 46 | 60 |
| **Aug** | 32.6 | 23.6 | 37.3 | 4.09 | 8.85 | 90 | 68 | 79 |
| **Sep** | 31.5 | 20.0 | 27.1 | 3.29 | 4.28 | 89 | 69 | 79 |
| **Oct** | 29.5 | 13.3 | 36.3 | 3.3 | 0.1 | 85 | 47 | 66 |
| **Nov** | 24.2 | 7.4 | 37.4 | 2.1 | 0.1 | 90 | 55 | 73 |
| **Dec** | 28.4 | 14.59 | 41.01 | 3.97 | 4.19 | 81 | 53 | 67 |
